# Orlando Protocol for single session ductal clearance of common bile duct stones at endosopic retrograde cholangiopancreatography

**DOI:** 10.1111/den.14719

**Published:** 2023-12-11

**Authors:** Ji Young Bang, C. Mel Wilcox, Udayakumar Navaneethan, Robert Hawes, Shyam Varadarajulu

**Affiliations:** ^1^ Digestive Health Institute, Orlando Health Orlando FL USA

**Keywords:** bile duct stones, ERCP, mechanical lithotripsy, single‐operator cholangioscopy, sphincteroplasty

## Abstract

**Objectives:**

Approach to management of common bile duct stones (CBDS) by endoscopic retrograde cholangiopancreatography (ERCP) is not standardized. We examined outcomes by applying predetermined protocol for CBDS management.

**Methods:**

When standard extraction techniques failed at ERCP, presence of tapered bile duct and stone–duct ratio were calculated. Large balloon sphincteroplasty (LBS) and/or mechanical/single‐operator cholangioscopy‐guided lithotripsy was performed based on presence of tapered bile duct and stone–duct mismatch. Primary outcome was single‐session ductal clearance. Secondary outcome was adverse events.

**Results:**

Of 409 patients treated over 16 months, 321 (78.5%) had no tapered bile duct or stone–duct mismatch, and single‐session ductal clearance was achieved using standard techniques in 99.7% over median duration of 14 min (interquartile range [IQR] 9–21 min). Of 88 (21.5%) patients with difficult CBDS, tapered duct was seen in 79 (89.8%) and/or stone–duct mismatch in 36 (40.9%). Single‐session ductal clearance was achieved in all 88 patients (100%) by LBS in 79 (89.8%), mechanical lithotripsy in 20 (22.7%), and single‐operator cholangioscopy‐guided lithotripsy in 16 (18.2%) over a median duration of 29 min (IQR 17–47 min). Overall, single‐session ductal clearance was achieved in 99.8% with adverse events in 17 (4.2%) that included perforation in two, postsphincterotomy bleeding in one, and mild/moderate post‐ERCP pancreatitis in 14 patients.

**Conclusions:**

A predetermined protocol optimized outcomes by enabling single‐session ductal clearance of CBDS with high technical success and low adverse events.

## INTRODUCTION

First‐line treatment option for common bile duct stones (CBDS) is endoscopic retrograde cholangiopancreatography (ERCP) with biliary sphincterotomy and stone extraction using standard devices, such as retrieval balloons and baskets. However, 15–20% of CBDS are difficult and not amenable to extraction by standard measures.^1^ These patients require advanced maneuvers mainly based on two technical concepts: dilation of papillary orifice (large balloon sphincteroplasty [LBS]) and/or fragmentation (lithotripsy) to facilitate stone extraction.^2^ European Society of Gastrointestinal Endoscopy recommends LBS as first‐line approach to treat difficult bile duct stones and reserves stone fragmentation techniques, mechanical lithotripsy (ML) or single‐operator cholangioscopy‐guided lithotripsy (SOCL) when LBS is unsuccessful.^3^ American Society for Gastrointestinal Endoscopy does not prioritize approaches but rather recommends either method.^2^ However, not all patients with difficult bile duct stones have a narrowed ampullary orifice that requires LBS or stone–duct mismatch that warrants lithotripsy. Moreover, given significant variations in technical and technological complexity and costs, application of lithotripsy techniques, ML vs. SOCL, should be more evidence‐based and tailored.

Although CBDS are relatively common indication for ERCP, because of a lack of standardization, procedures can become prolonged and inefficient, leading to use of multiple devices, techniques, and adoption of alternate treatment modalities.^4^, ^5^, ^6^, ^7^ This not only leads to multiple reinterventions but also makes procedures costlier. An internal audit of 500 consecutive ERCP procedures performed at our institution revealed single‐session ductal clearance in 92.6% with reinterventions primarily in patients with difficult stones. To address this limitation, we recently conducted a randomized trial in which SOCL was found to be superior to LBS when stone size exceeded diameter of bile duct distal to the stone and LBS alone was less likely to be effective as the sole maneuver when large CBDS are associated with tapered bile duct.^8^ We integrated these findings to develop the Orlando Protocol for management of CBDS. Our hypothesis was that by following this standardized protocol, single‐session ductal clearance could be achieved in majority of patients, particularly those with difficult CBDS.

## MATERIALS AND METHODS

### Patients and settings

As part of the quality improvement process, a management protocol was applied prospectively on consecutive patients with CBDS aged ≥18 years who underwent ERCP over 16 months from February 2022 to May 2023 at Orlando Health (Orlando, FL, USA). All procedures were performed by four therapeutic endoscopists (J.Y.B., U.N., R.H., S.V.) without trainee participation. Patients with intrahepatic ductal stones, altered surgical anatomy, pancreaticobiliary malignancy, benign biliary stricture, pregnancy, thrombocytopenia, irreversible coagulopathy, and anticoagulation or antiplatelet therapy that could not be discontinued were excluded. Written informed consent was obtained from all patients undergoing ERCP. Because data were collected for quality improvement, after administrative review of data collection tools (Appendix S1), the institutional review board waived requirement for informed consent and ethical approval was obtained for data analysis (approval notice number 1941655). All authors had full access to study data and have reviewed and approved the final manuscript.

### Management protocol

**Figure 1:**
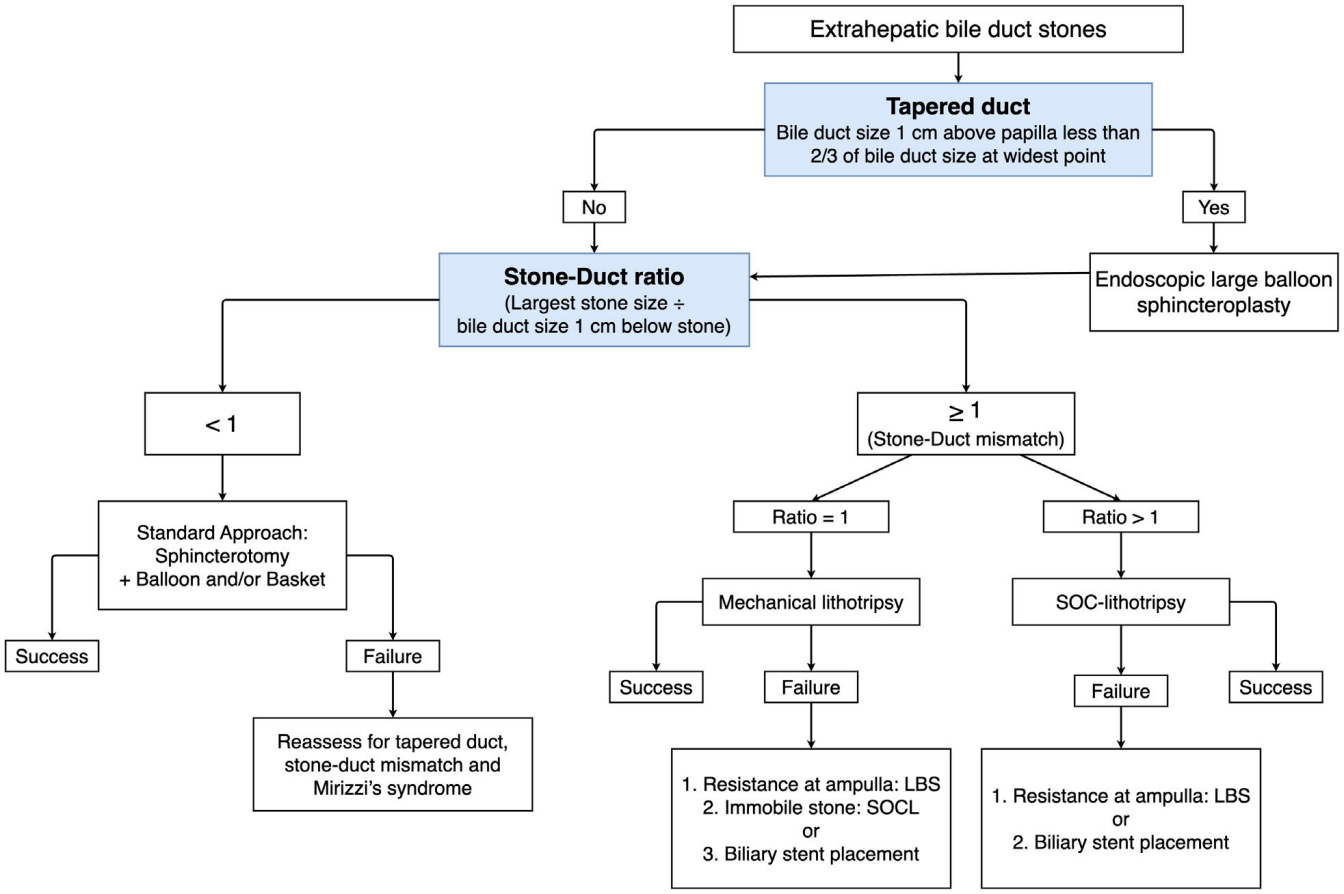
Orlando Protocol for management of common bile duct stones. LBS, large balloon sphincteroplasty; SOCL, single‐operator cholangioscopy‐guided lithotripsy.

Common bile duct stones were managed adopting Orlando Protocol (Fig. 1).

#### Standard approach

**Figure 2:**
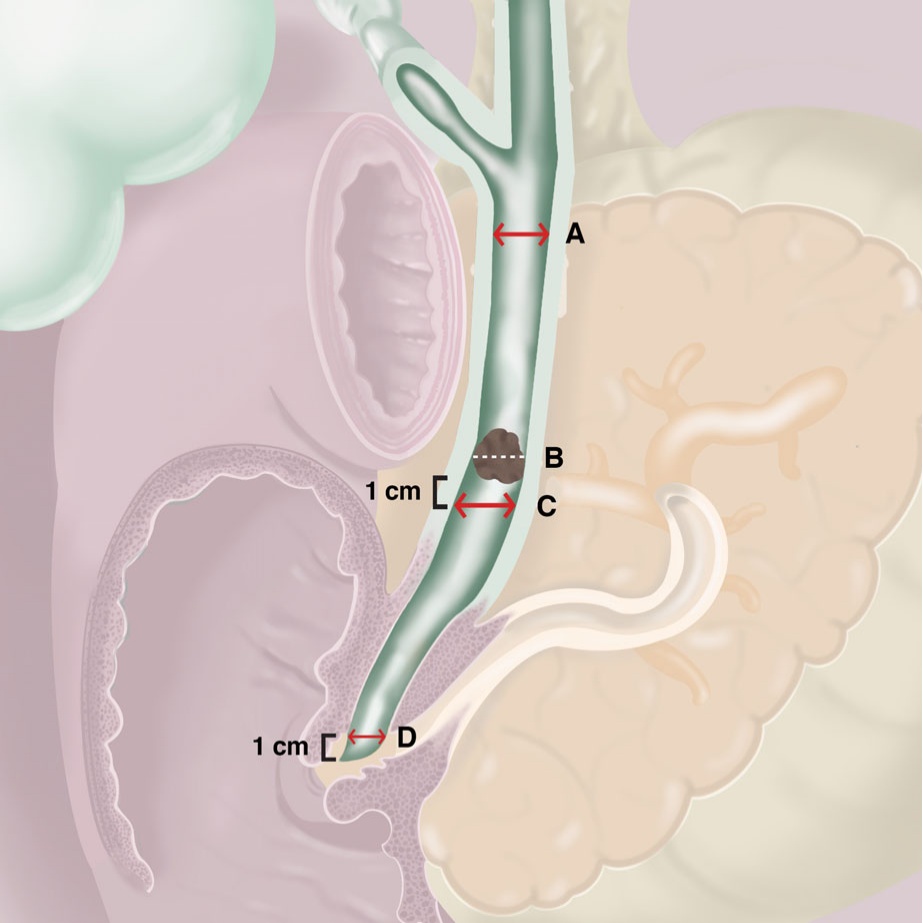
Illustration depicting areas of measurement for stone size and bile duct diameter. (A) Largest bile duct diameter. (B) Largest stone size. (C) Bile duct diameter 1 cm below the stone. (D) Distal bile duct diameter 1 cm above the ampullary orifice.

All interventions were performed using a therapeutic duodenoscope under general anesthesia with patients in prone position. In patients who had undergone previous ERCP, biliary endoprosthesis, if present, was removed using a snare. Bile duct was selectively cannulated using wire‐guided sphincterotome and opacified using contrast. Diameter of largest stone, diameter of common bile duct (CBD) 1 cm distal to largest stone, diameter of widest area of CBD, and diameter of CBD 1 cm above ampullary orifice were measured intraprocedurally (Video S1) with diameter of duodenoscope shaft as reference standard to determine stone–duct ratio and presence of tapered bile duct (Fig. 2). Measurements were made using software inbuilt within fluoroscopy system (E‐View.AI.; Omega Imaging, Sanford, FL, USA). In patients with native papilla or inadequate papillotomy, biliary sphincterotomy was performed up to duct–duodenal junction. Stone retrieval was attempted, based on duct size, using balloons ranging from 8.5 to 20 mm in diameter (Multi‐3V Plus Extraction Balloons; Olympus, Tokyo, Japan) and/or Dormia basket (Olympus) (Video S1).

#### Approach to difficult stones

**Figure 3:**
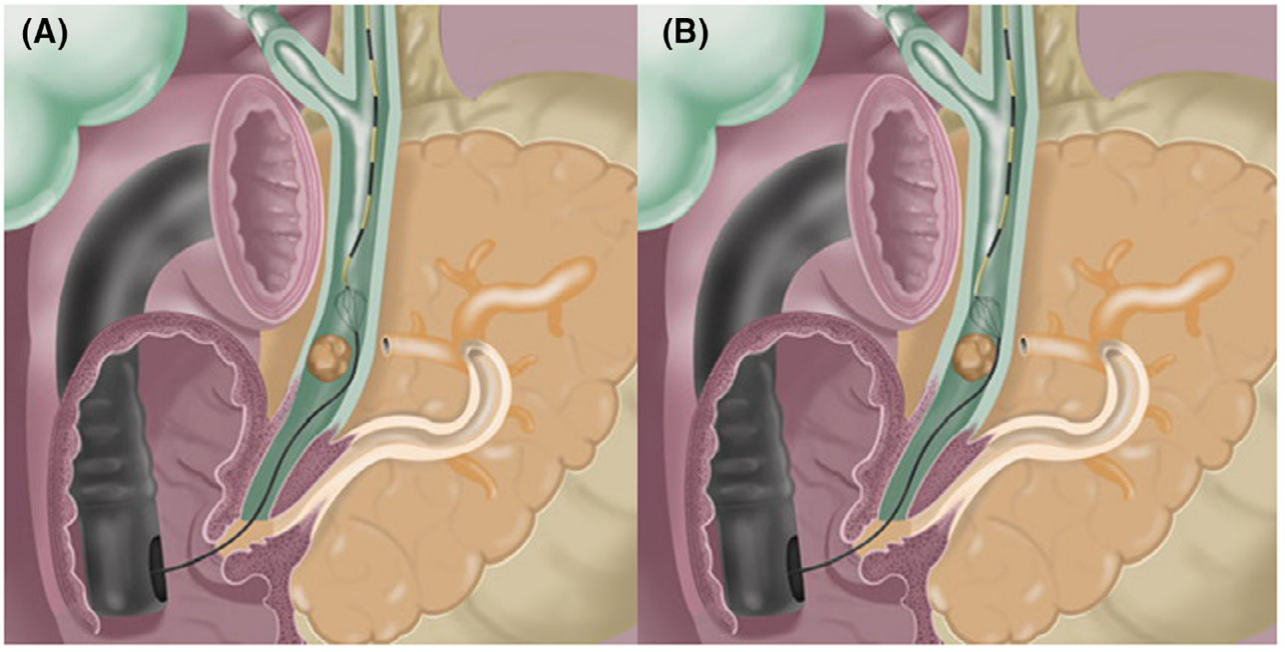
(A) Illustration depicting the passage of a mechanical lithotripter adjacent to a common bile duct stone with ability to fully open the basket for stone entrapment. (B) Illustration depicting the fulcrum effect induced by an impacting stone in the common bile duct that precludes opening of the basket for stone entrapment.

Large balloon sphincteroplasty was undertaken in patients with tapered bile duct, intraductal lithotripsy in patients with stone–duct mismatch, and LBS followed by intraductal lithotripsy in patients who had both tapered bile duct and stone–duct mismatch. Choice of ML or SOCL was determined by degree of stone–duct mismatch. ML was performed when stone size was equivalent to bile duct diameter that corresponded to stone–duct ratio of 1. SOCL was performed when size of stone exceeded bile duct diameter that corresponded to ratio >1. This determination was made by review of fluoroscopic images and endoscopic recordings from a previous randomized trial where we observed that ML could be passed adjacent to a stone and the basket fully opened when stone–duct ratio was ≤1; however, when ratio was >1, the fulcrum effect induced by the stone precluded basket opening for stone entrapment (Fig. 3).^8^

##### Large balloon sphincteroplasty

Major duodenal papilla was dilated using radial expansion balloon with size range 8–20 mm depending on distal CBD diameter (Video S1) (further information in Appendix S1). Balloon was gradually filled with diluted contrast medium under endoscopic and fluoroscopic guidance to observe gradual disappearance of waist in the balloon, which was taken to indicate progressive dilation of the orifice. Balloon was kept inflated for 60 s. After balloon deflation, stone extraction was attempted using retrieval balloon or basket. Intraductal lithotripsy was performed when stone extraction was unsuccessful.

##### Intraductal lithotripsy

###### Single‐operator cholangioscopy‐guided lithotripsy

Cholangioscope (SpyGlass DS; Boston Scientific, Marlborough, MA, USA) was advanced through accessory channel and cannulation was achieved using free‐hand technique (Video S1). Device was positioned en face to the most distal stone. Lithotripsy using Holmium laser (Lumenis, energy settings 1 J, 10 Hz; Boston Scientific, Marlborough, MA, USA) or electrohydraulic technique (Autolith Touch, titrated shot frequency 1–30/s and number 1–60; Boston Scientific, Marlborough, MA, USA) was performed by insertion of a probe through biopsy channel of cholangioscope. Choice of modality was determined by discretion of endoscopist. Lithotripsy was continued until all fragmented stones were small enough to be retrieved using basket or balloon.

###### Mechanical lithotripsy

This was performed by passing lithotripsy catheter (Trapezoid, Boston Scientific; size 1.5–3 cm) proximal to stone. Basket size was tailored to size of stone and diameter of bile duct. Once basket was fully opened, it was slowly withdrawn to entrap stone within the mesh and then lithotripsy was applied to fragment the stone (Video S1).

Because ductal clearance can sometimes be challenging in presence of numerous small stone fragments lodged in distal bile duct after intraductal lithotripsy, LBS tailored to size of distal bile duct was performed to facilitate stone extraction when such resistance was encountered at ampullary orifice.

##### Long‐term stenting

When treatment failed, 10F straight or double pigtail plastic stent(s) were placed for biliary decompression with follow‐up ERCP at 30 days, with method of stone extraction left to endoscopist discretion. Patient follow‐up can be seen in Appendix S1.

### Outcome measures

Primary outcome measure was treatment success, which was defined as ability to achieve ductal clearance in one session. Ductal clearance was defined as lack of filling defects in cholangiogram obtained using fully inflated occlusion balloon at conclusion of procedure, in conjunction with absence of reinterventions for bile duct stones until 6 months after intervention. Secondary outcome measure was adverse events, which was graded by established criteria^9^ (Appendix S1).

### Definitions

Difficult bile duct stones were defined as stones in which attempts at extraction after biliary sphincterotomy using retrieval balloon and/or basket were unsuccessful. Stone–duct ratio was defined as size of largest stone divided by diameter of extrahepatic bile duct 1 cm distal to stone (Fig. 2). Tapered bile duct was defined as ratio of diameter of distal bile duct, measured 1 cm above ampullary orifice, to the widest point in proximal CBD being less than two‐thirds (0.66) (Fig. 2). Procedural duration was defined as time from duodenoscope insertion to its withdrawal.

### Statistical analysis

Continuous variables were summarized as means with standard deviations and median with interquartile ranges (IQRs) and compared between approaches using Wilcoxon rank‐sum test or Student' *t*‐test as indicated. Categorical variables were summarized as frequencies and proportions and compared using Fisher's exact test or χ^2^‐test. Because of occurrence of separation with logistic regression analysis, penalized logistic regression with Firth correction was performed to identify predictors for choice of lithotripsy technique, ML vs. SOCL, for treatment of difficult CBDS.^10^ Statistical significance was determined as *P* < 0.05 (Appendix S1).

## RESULTS

Of 462 patients who underwent ERCP for CBDS from February 2022 to May 2023, 53 were excluded because of altered anatomy (*n* = 18), coexisting benign/malignant biliary stricture (*n* = 22), intrahepatic stones (*n* = 2), anticoagulation (*n* = 5), children (*n* = 5), or pregnancy (*n* = 1). Baseline characteristics of 409 patients who formed study cohort are shown in Table 1. Mean age of study cohort was 61.9 years (SD 20.3), women constituted 56.5%, and 18.6% had previously failed CBDS extraction at outside facilities. Biliary access was successful in all patients, with 6.8% requiring advanced cannulation techniques.

**Table 1:** Baseline characteristics of 409 patients who formed the study cohort

	All patients (*n* = 409)
Age (years)	
Mean (SD)	61.9 (20.3)
Median	67
IQR	48–78
Gender, *n* (%)	
Female	231 (56.5)
Male	178 (43.5)
Prior failed ERCP, *n* (%)	76 (18.6)^†^
Largest stone diameter (mm)	
Mean (SD)	7.3 (3.9)
Median	6
IQR	5–9
Multiple stones, *n* (%)	312 (76.3)
Largest bile duct diameter (mm)	
Mean (SD)	10.7 (3.2)
Median	10
IQR	9–12
Tapered bile duct present, *n* (%)	79 (19.3)
Stone–duct ratio	
Mean (SD)	0.67 (0.22)
Median	0.67
IQR	0.50–0.80
Administration of rectal indomethacin, *n* (%)^‡^	159 (38.9)
Stone–duct mismatch present, *n* (%)	36 (8.8)
Advanced technique required for cannulation, *n* (%)^§^	28 (6.8)
Prior biliary sphincterotomy, *n* (%)^¶^	76 (18.6)
Extension of prior biliary sphincterotomy^††^	60 (78.9)
Advanced technique used, *n* (%)	88 (21.5)
Balloon sphincteroplasty, *n* (%)	79 (19.3)
Mechanical lithotripsy, *n* (%)	20 (4.9)
SOC‐guided lithotripsy, *n* (%)^‡‡^	16 (3.9)
Total procedure duration (min)	
Mean (SD)	21.8 (19.1)
Median	15.5
IQR	11–26
Adverse events, *n* (%)^§§^	17 (4.2)
Admission postprocedure, *n* (%)^§§^ ^,^ ^¶¶^	23 (5.6)

^†^ In one patient, biliary stent was placed without removal of difficult stone at index endoscopic retrograde cholangiopancreatography (ERCP) because of cholangitis and septic shock.

^‡^ Rectal indomethacin was administered when advanced cannulation techniques were practiced or when cannulation exceeded eight attempts.

^§^ Advanced technique includes pancreatic stent placement, precut sphincterotomy.

^¶^ Biliary sphincterotomy performed at outside facilities before referral.

^††^ Applicable only in patients with prior biliary sphincterotomy (denominator 76).

^‡‡^ Electrohydraulic lithotripsy in 13, Holmium laser in three.

^§§^ In one patient with duodenal perforation, the perforation was successfully treated with placement of an over‐the‐scope clip, which sealed the perforation and confirmed on fluoroscopy. During hospitalization postprocedure, the patient aspirated clear liquids, developed aspiration pneumonia, and subsequently had a cardiopulmonary arrest. Supportive care from this patient was withdrawn 7 days after intervention.

^¶¶^ Admission postprocedure to the treating facility because of acute pancreatitis (*n* = 9), perforation (*n* = 2), abdominal pain (*n* = 11), bleeding (*n* = 1).

IQR, interquartile range; SD, standard deviation; SOC, single‐operator cholangioscopy.

### Standard approach to bile duct stones

A total of 321 of 409 (78.5%) patients had no tapered duct or stone–duct mismatch. Median size of largest stone was 6 mm (IQR 5–7 mm), median stone–duct ratio was 0.60 (IQR 0.50–0.71), and median procedural duration was 14 min (IQR 9–21 min) (Table 2). Single‐session ductal clearance was achieved using standard techniques in 320 of 321 (99.7%) patients. Duodenal perforation was encountered in two patients during duodenoscope navigation that were managed endoscopically using over‐the‐scope clips (Ovesco Endoscopy USA Inc., Cary, NC, USA). A 10F plastic biliary stent was placed in one patient who underwent a successful second ERCP for stone extraction. In a second patient, although complete stone retrieval was achieved during a single ERCP session, she died from aspiration pneumonia and septic shock. Mild and moderate pancreatitis were encountered in six and three patients, respectively, who were managed conservatively.

**Table 2:** Procedural details and technical outcomes of 409 patients treated by standard approach and those with difficult stones managed by advanced techniques

	Standard approach (*n* = 321)	Advanced techniques (*n* = 88)	*P*‐value
Age (years)			
Mean (SD)	59.5 (20.8)	70.5 (15.5)	<0.001
Median	65	73.5	
IQR	46–76	61.5–82	
Gender, *n* (%)			
Female	182 (56.7)	49 (55.7)	0.865
Male	139 (43.3)	39 (44.3)	
Largest stone diameter (mm)			
Mean (SD)	6.0 (2.4)	12.0 (4.5)	<0.001
Median	6	12	
IQR	5–7	9–15	
Largest bile duct diameter (mm)			
Mean (SD)	10.0 (2.5)	13.2 (3.9)	<0.001
Median	10	12	
IQR	8–11	10–15	
Stone–duct ratio			
Mean (SD)	0.60 (0.18)	0.94 (0.18)	<0.001
Median	0.60	0.90	
IQR	0.50–0.71	0.80–1.03	
Total procedure duration (min)			
Mean (SD)	18.4 (16.8)	34.6 (22.0)	<0.001
Median	14	29	
IQR	9–21	17–47	
Adverse events, *n* (%)	11 (3.4)	6 (6.8)	0.222

IQR, interquartile range; SD, standard deviation.

### Approach to difficult bile duct stones

Of 88 (21.5%) patients with difficult CBDS, tapered duct was seen in 79 (89.8%) and stone–duct mismatch in 36 (40.9%). Of 79 patients with tapered duct, stone–duct mismatch was not observed in 52. Of 36 patients with stone–duct mismatch, tapered duct was not observed in 9. Median size of largest stone was 12 mm (IQR 9–15 mm) and median stone–duct ratio was 0.90 (IQR 0.80–1.03) (Table 2). Single‐session ductal clearance was achieved in all 88 patients by LBS in 79 (89.8%), ML in 20 (22.7%), and SOCL in 16 (18.2%) over a median duration of 29 min (IQR 17–47 min).

All 36 patients with stone–duct mismatch were successfully treated using either ML or SOCL and did not require both techniques for achieving ductal clearance; nine patients without tapered duct did not require LBS. Of 79 patients with tapered bile duct, LBS was technically successful in all patients. None of the patients without concomitant stone–duct mismatch required adjunctive lithotripsy for achieving ductal clearance.

Adverse events were encountered in six patients that included postsphincterotomy bleeding in one, mild pancreatitis in four, and moderate pancreatitis in one who were all managed conservatively.

### Outcome measures

**Figure 4:**
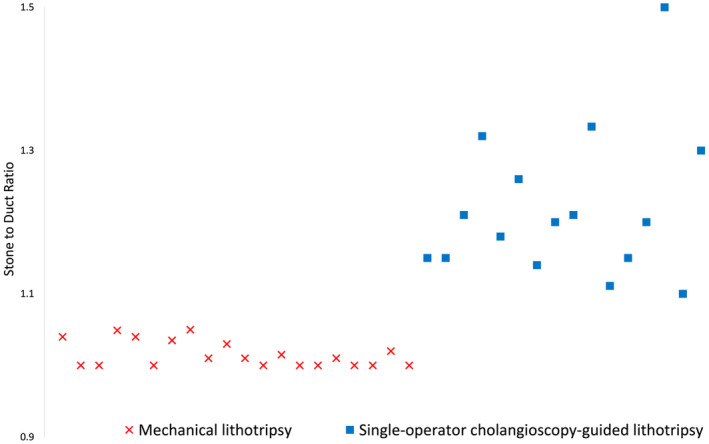
Scatter plot to show the relationship between the stone–duct ratio and need for mechanical lithotripsy or single‐operator cholangioscopy‐guided lithotripsy.

By adopting protocol‐based approach, single‐session ductal clearance was achieved in 99.8% (408 of 409) patients with adverse events in 4.2% (17 of 409) that were severe in one (perforation with aspiration pneumonia), moderate in five (perforation 1, pancreatitis 4), and mild (bleeding 1, pancreatitis 10) in 11. Further information on regression analysis in Table S1 and Figure 4.

## DISCUSSION

Despite encountering technical challenges not infrequently at ERCP,^11^ there are no standardized approaches to management of CBDS. Proposed protocol provides a framework whereby the underlying challenge is clearly defined and then objectively managed. The treatment approach facilitated single‐session ductal clearance in more than 99% of patients with adverse events in less than 5%.

European Society of Gastrointestinal Endoscopy guidelines recommend empiric LBS as the first step in all patients with difficult CBDS and then to perform intraductal lithotripsy as deemed appropriate.^3^ By performing LBS only in patients with tapered bile duct or when resistance was encountered at level of major duodenal papilla, we obviated need for papillary dilation along with its attendant risks and costs in 10% of patients with difficult bile duct stones. Despite practice of ML and SOCL being existent for more than 30 and 15 years, respectively, there are no definitive recommendations on when either technique should be prioritized at ERCP.^2^, ^3^ Both techniques are challenging, labor intensive, and SOCL in particular is more expensive.^6^, ^8^ We observed that when stone–duct ratio was 1, it was technically feasible to perform ML with successful treatment outcomes in all patients. As the ratio increased, ML was unsuccessful. Therefore, for stone–duct ratio greater than 1, we recommend prioritizing SOCL over ML as odds for successful stone extraction are 35 times greater. This observation has previously been reported in a large retrospective study from India where degree of impaction, and not stone size, was found to be an independent predictor of technical failure for ML.^12^ Although we did not perform a cost analysis, both SOCL and laser/electrohydraulic procedures are significantly more expensive than ML. Costs for commonly used SOC device (SpyGlass; Boston Scientific), electrohydraulic lithotripsy probe, and ML (Trapezoid; Boston Scientific) are US $2400, $430, and $250, respectively.

Another important observation is worth highlighting. In our study cohort, 59.2% of patients referred after a failed ERCP for management of difficult stones could be treated at our center by performing standard maneuvers only. Oftentimes, stone extraction fails not because of stone–duct mismatch or tapered bile duct but because sphincterotomy was inadequate, which could make extraction maneuvers difficult. In our experience, by performing biliary sphincterotomy to the duct–duodenal junction and withdrawing a fully inflated retrieval balloon in axis of duodenoscope, a significant proportion of stones deemed difficult can be successfully retrieved without advanced maneuvers.

**Figure 5:**
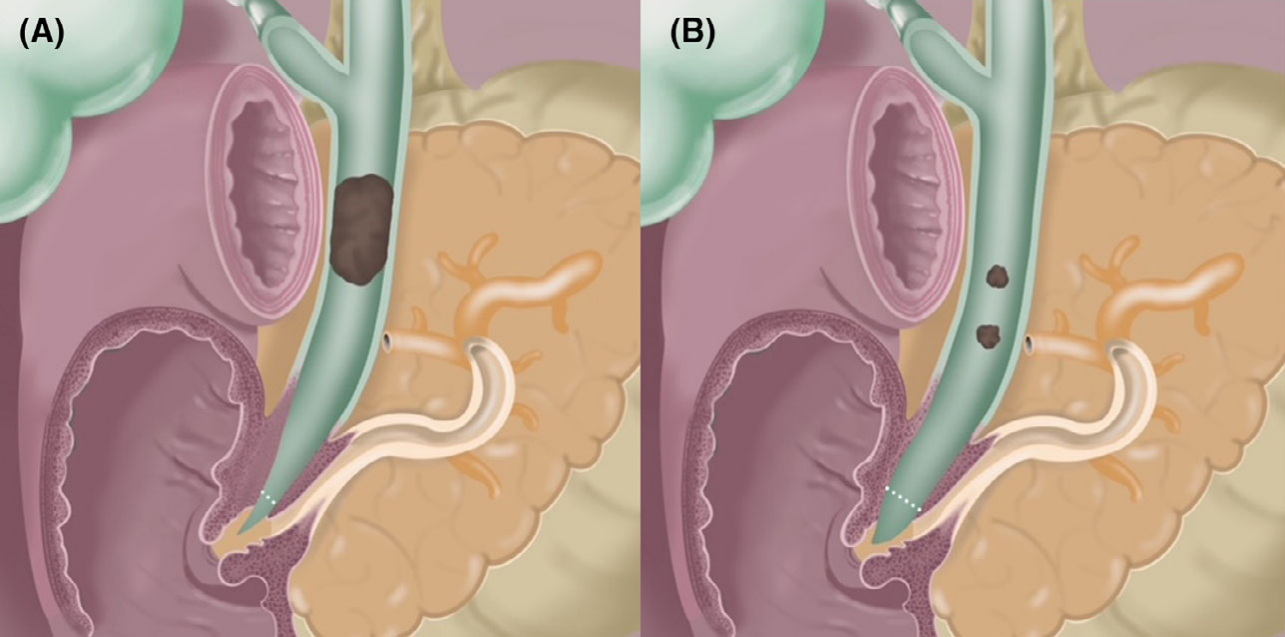
(A) Illustration depicting a tapered bile duct where distally the duct is configured as a “V,” as compared with (B) the “U” shape of a nontapered bile duct.

What lessons can be learned from this study that can affect the practice of CBDS management? First, when standard stone retrieval maneuver fails at ERCP, it is important to ascertain presence of tapered bile duct and stone–duct ratio. Based on findings of our prior randomized trial,^8^ we determined that when distal bile duct is less than two‐thirds (66%) of diameter of the widest extrahepatic CBD, performing LBS alone may facilitate successful stone extraction, particularly when there is no stone–duct mismatch. From a practical standpoint, if distal duct is shaped as a “V” rather than a “U,” it is likely tapered and will benefit from LBS (Fig. 5). Second, in patients with impacted stones, when stone–duct ratio is 1, ML alone can facilitate successful stone retrieval. When ratio is >1, SOCL is indispensable for successful outcomes. Although it may be challenging at times to make precise calculations intraprocedurally, a practical method to determine this cut‐off is at ductal opacification. On fluoroscopic view, if a stone is seen occupying the bile duct in its entirety but the injectant contrast medium can opacify the upstream duct and retrieval balloon catheter can be advanced proximally without any resistance, the ratio is generally 1. Alternatively, if contrast injection is performed under pressure and resistance is encountered on passage of a retrieval balloon catheter proximal to the stone, ratio is likely >1 (Fig. 3). By performing this thoughtful assessment, patients can be appropriately triaged, thereby facilitating optimal clinical outcomes and avoiding expensive, unnecessary, or repeat interventions. Third, in patients with difficult CBDS who had both tapered bile duct and stone–duct mismatch, performing LBS first enables easy passage of lithotripsy devices and subsequent stone retrieval becomes less cumbersome. Fourth, by performing adequate sphincterotomy and precisely executing standard stone retrieval maneuvers, significant proportion of stones deemed “difficult” can be successfully extracted. Finally, although patients with CBDS are inherently different with a unique set of challenges and the protocol may not be uniformly applicable to all situations, we believe that it can serve as a roadmap enabling a tailored treatment strategy, which leads to improved clinical outcomes and cost‐effective care.

There are several limitations to this study. One, this was a prospective observational study and not a randomized trial comparing treatment approaches. However, compared with reintervention rate of 7.4% before protocol implementation in our practice, and 5–10% at other expert centers,^13^, ^14^ outcomes for single session ductal clearance reported in this study were more robust. Two, although stone–duct assessments were made using an inbuilt fluoroscopy software, this could be tailored to local needs using diameter of duodenoscope shaft as reference standard. Three, study findings are applicable only to CBDS and not for patients with intrahepatic stones or other concomitant pathology. Four, comparative studies with standard stone retrieval methods to assess treatment outcomes will be beneficial. Five, given study design (protocol validation), subjects were allocated to treatment type based on cholangiogram findings, with potential risk of selection bias.

In conclusion, proposed protocol enables efficient single‐session ductal clearance of CBDS with high rate of technical success and low adverse events. Consideration should be given to incorporating this protocol in treatment guidelines and in ERCP teaching curriculum because it is likely to improve clinical outcomes and yield cost savings.

## CONFLICT OF INTEREST

Author J.Y.B.: consultant for Olympus America Inc. and Boston Scientific Corporation. S.V.: consultant for Boston Scientific Corporation, Olympus America Inc., and Medtronic. R.H.: consultant for Olympus America Inc, Fujifilm, GIE Medical. U.N.: consultant for Janssen, Pfizer, Takeda, AbbVie, Bristol Myers Squibb, and GIE Medical Inc. C.M.W. declares no conflict of interest for this article.

## FUNDING INFORMATION

None.

## Supporting information


**Appendix S1** Additional information on methods, results, and prospective data collection tool based on the Orlando Protocol for patients undergoing endoscopic retrograde cholangiopancreatography for common bile duct stones.
**Table S1** Multiple logistic regression examining the factors associated with choice of lithotripsy technique for cohort of patients with stone–duct mismatch.

**Video S1** Demonstration of the Orlando Protocol for management of common bile duct stones.
