# Supplementary material for: Orlando Protocol for single session ductal clearance of common bile duct stones at endosopic retrograde cholangiopancreatography
Source: Dig Endosc. 2023 Dec 11;36(7):825–33. doi: 10.1111/den.14719 (PMC12136243; doi:10.1111/den.14719)
Supplement: Supplementary file 1 — Appendix S1 Additional information on methods, results, and prospective data collection tool based on the Orlando Protocol for patients undergoing endoscopic retrograde cholangiopancreatography for common bile duct stones. Table S1 Multiple logistic regression examining the factors associated with choice of lithotripsy technique for cohort of patients with stone–duct mismatch. [file DEN-36-825-s002.pdf]

Supplementary Appendix

ORLANDO PROTOCOL FOR SINGLE SESSION DUCTAL CLEARANCE OF COMMON BILE DUCT STONES AT ERCP

Ji Young Bang MD MPH, C. Mel Wilcox MD, Udayakumar Navaneethan MD,
Robert Hawes MD, Shyam Varadarajulu MD

Methods - Large balloon sphincteroplasty

In patients with a tapered bile duct but without ductal dilation, sphincteroplasty was still undertaken using a smaller balloon that was tailored to the bile duct diameter to facilitate stone extraction and easy passage of accessories.

Methods - Outcome measures

All data were prospectively entered in an electronic database following each procedure. Per unit policy, all patients were discharged home on the same day if they met discharge criteria (clinically stable without new or worsening of existing symptoms). Once discharged home, per unit policy, patients were contacted by telephone calls within one week post-procedure to assess for adverse events. Thus, information on adverse events including acute pancreatitis was captured in all subjects. All patients with new or worsening of existing symptoms post-procedure were hospitalized per standard of care and pancreatic enzymes (amylase and lipase) were measured within 24 hours when indicated to evaluate for acute pancreatitis. Hospital records were reviewed in all patients who were hospitalized for management of adverse events and acute pancreatitis was defined according to the widely validated consensus definition of new onset or worsening of pain in the upper abdomen, with an elevation in pancreatic enzymes of at least three times the upper limit of the normal at 24 hours after the procedure.

Methods - Statistical Analysis

Patient characteristics, procedural details, and treatment outcomes were summarized and compared when appropriate between standard treatment approach and approach to difficult stones.

Due to occurrence of separation with logistic regression analysis, penalized logistic regression with Firth's correction was performed to identify predictors for choice of lithotripsy technique, ML versus SOCL, for treatment of difficult CBDS. Clinically relevant variables, namely patient demographics, presence of tapered bile duct, ratio of stone to extrahepatic bile duct diameter and multiple stones were taken as predictor variables for inclusion in the analysis. Statistical significance was determined as p-

value <0.05. Datasets were compiled using Microsoft Excel (Microsoft Corporation, Richmond, WA), and analyses were performed using Stata version 17 (StataCorp LP, College Station, TX).

Results - Logistic regression analysis

On multiple logistic regression analysis, there was significant association between degree of stone-duct mismatch and choice of lithotripsy technique whereby stone-duct ratio >1 was significantly associated with need for single operator cholangioscopy-guided lithotripsy (odds ratio [OR] 35.7, 95% CI, 2.57-495.3, p=0.008).

Supplementary Table 1. Multiple logistic regression analysis examining the factors associated with the need for single-operator cholangioscopy-guided lithotripsy for cohort of patients with stone-duct mismatch

**Variable**	**Odds ratio**	**95% CI**	**p-value**
**Age: ≥ 70 vs. < 70 years**	1.82	0.35 - 9.59	0.480
**Gender: Male vs. Female**	1.03	0.19 - 5.49	0.972
**Stone-Duct ratio: >1 vs. 1**	35.7	2.57 - 495.3	0.008
**Presence of a tapered duct: Yes vs. No**	1.59	0.25 - 10.2	0.622
**Multiple stones: Yes vs. No**	0.47	0.0023 - 96.5	0.780

Abbreviations: CI, confidence interval

Title	Registry of patients undergoing endoscopic management of bile duct stones	
Subject ID:	STO-___ ___ ___	Principal Investigator: Ji Young Bang, MD

Date of procedure: ___________ / ____________ / ____________

Demographics

Age: ______________________

Gender: Male: ☐ Female: ☐

Race: Caucasian: ______ Black: _______ Hispanic: _______ Asian: _______

Other: _________________________________

Status: Inpatient: ☐ Outpatient: ☐

History

Imaging performed showing bile duct stone: None ☐ CT ☐ MRI/MRCP ☐

Prior ERCP attempt for stone removal: No ☐ Yes ☐

If yes, date of ERCP: ___________________________________

If yes, prior technique for stone removal:

Basket ☐ Balloon ☐ Mechanical Lithotripter ☐ SOC-guided lithotripsy ☐

Biliary stent in place from prior ERCP for stone removal: No ☐ Yes ☐

Reason for failure of stone removal at last session if applicable:

Large stone ☐ Impacted stone ☐ Multiple stones ☐

Other: ☐ ____

____

Prior cholecystectomy: no ☐ yes ☐

Notes: ____

____

____

____

____

____

Title	Registry of patients undergoing endoscopic management of bile duct stones	
Subject ID:	STO-___ ___ ___	Principal Investigator: Ji Young Bang, MD

Stone removal maneuvers performed per algorithm (Circle all that apply):

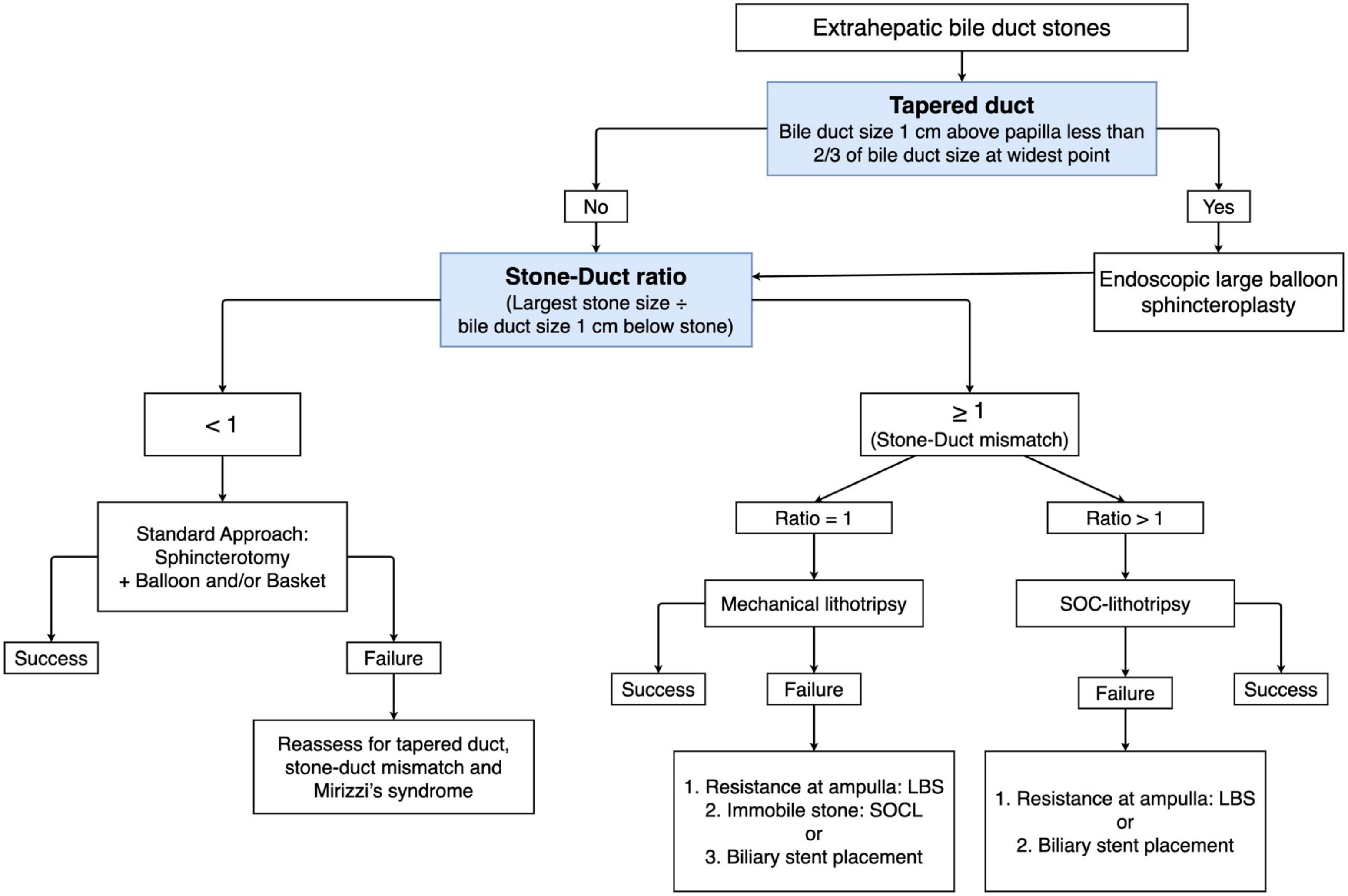

Title	Registry of patients undergoing endoscopic management of bile duct stones	
Subject ID:	STO-___ ___ ___	Principal Investigator: Ji Young Bang, MD

Stone management algorithm followed: Yes ☐ No ☐

If not followed, reason: ____

____

____

Stone characteristics on cholangiogram

Size of largest bile duct stone (B): _______________ mm

Location of largest bile duct stone: Bottom 50% of bile duct ☐ Top 50% of bile duct ☐

Total no. of bile duct stones: _____________________

Bile duct characteristics on cholangiogram

Size of bile duct when measured at its widest point (A): _______________ mm

Size of bile duct 1cm distal to the largest stone (C): _______________ mm

Size of distal bile duct 1cm above ampullary orifice (D): _______________ mm

Ratios

Stone:Duct ratio = Largest stone size ÷ bile duct size 1cm distal to the largest stone (B/C):

____

Duct tapering = Distal bile duct size 1cm above ampullary orifice ÷ Bile duct size at its widest point (D/A):

____

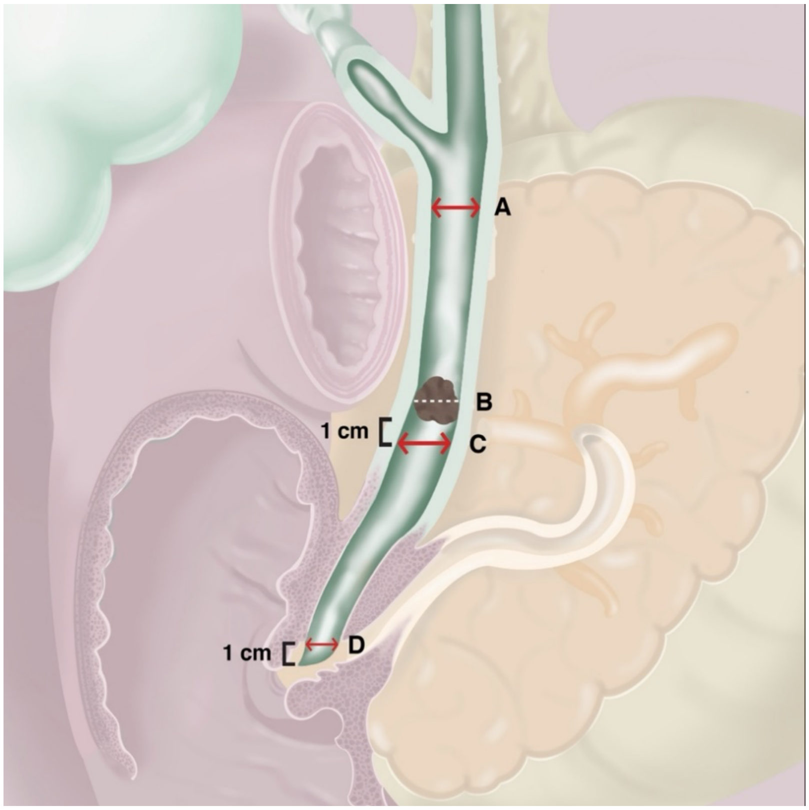

Title	Registry of patients undergoing endoscopic management of bile duct stones	
Subject ID:	STO-___ ___ ___	Principal Investigator: Ji Young Bang, MD

Details on techniques used (check all that apply)

1. **Biliary sphincterotomy:** Yes ☐ No ☐
   Periampullary diverticulum present: Yes ☐ No ☐
   Evidence of prior sphincterotomy: Yes ☐ No ☐
   Size of biliary sphincterotomy performed: _______________ mm

2. **Large balloon sphincteroplasty:** Yes ☐ No ☐
   Size of balloon for dilation: _____________________ mm

3. **SOC-guided lithotripsy:** Yes ☐ No ☐
   Type of energy used: Laser ☐ EHL ☐

4. **Mechanical lithotripsy:** Yes ☐ No ☐
   Type of lithotripter used: Trapezoid ☐ Other ☐ - type _______________________
   Size of lithotripter used: _____________________ mm

5. **Duct sweep for stone removal:**
   1. Retrieval balloon used to sweep the bile duct: Yes ☐ No ☐
      If yes, Retrieval balloon successful in achieving ductal clearance: Yes ☐ No ☐
   2. Standard stone retrieval basket used to sweep the bile duct: Yes ☐ No ☐
      If yes, standard stone retrieval basket successful in achieving ductal clearance: Yes ☐ No ☐

6. **Biliary stent placement** as complete ductal clearance not achieved during this procedure:
   Yes ☐ No ☐

Total procedure duration: ________________ mins

Title	Registry of patients undergoing endoscopic management of bile duct stones	
Subject ID:	STO-___ ___ ___	Principal Investigator: Ji Young Bang, MD

Intraprocedural complication: ☐ Yes ☐ No

If yes, state:

☐ Aspiration ☐ Bleeding ☐ Cardiovascular ☐ Death

☐ Perforation ☐ Pneumoperitoneum ☐ Respiratory

☐ Other/give details:

____

____

____

Adverse event management (specify): ☐ Surgery ☐ IR ☐ Medical

Explain treatment measures: ____

____

____

____

Post-procedure length of hospital stay for adverse events: ________ days

Length of stay in ICU for adverse events: _________ days

Notes: ____

____

____

____

____

____

Title	Registry of patients undergoing endoscopic management of bile duct stones	
Subject ID:	STO-___ ___ ___	Principal Investigator: Ji Young Bang, MD

Procedure summary

1. STANDARD TECHNIQUE achieved ductal clearance: Yes ☐ No ☐

2. BALLOON SPHINCTEROPLASTY performed: Yes ☐ No ☐
   If yes, ductal clearance achieved after balloon sphincteroplasty: Yes ☐ No ☐

3. SOC performed: Yes ☐ No ☐
   If yes, SOC achieved ductal clearance: Yes ☐ No ☐

4. MECHANICAL LITHOTRIPSY performed: Yes ☐ No ☐
   If yes, mechanical lithotripsy achieved ductal clearance: Yes ☐ No ☐
